# A Review of Hodgkin Lymphoma in the Era of Checkpoint Inhibitors

**DOI:** 10.7759/cureus.41660

**Published:** 2023-07-10

**Authors:** Christopher J Schimmoeller, Craig Bastian, Jessica Fleming, Joshua Morales

**Affiliations:** 1 Internal Medicine, Carilion Clinic - Virginia Tech Carilion, Roanoke, USA; 2 Hematology and Oncology, Blue Ridge Cancer Care, Roanoke, USA

**Keywords:** cancer immunotherapy, hodgkin lymphoma, general radiation oncology, chemotherapy agents, checkpoint inhibitor therapy

## Abstract

Hodgkin lymphoma (HL) is a hematopoietic malignancy of B-cells that has a bimodal distribution with respect to age and incidence. With the introduction of doxorubicin (Adriamycin), bleomycin, vinblastine, and dacarbazine (ABVD) and radiation combined, the prognosis of HL has significantly improved, with five-year survival rates approaching 95%. While HL has become highly curable, the side effect profiles of ABVD are dire and warrant continuous review. Because HL is often diagnosed in populations in their 20s-30s, patients are forced to undergo fertility preservation procedures as well as deal with other long-term side effects of chemotherapy (including doxorubicin dose-dependent cardiotoxicity and bleomycin-induced lung toxicity). The opportunity cost of the treatment in the short term and vulnerability to treatment-induced malignancies decades later dramatically affect the quality of life of HL patients. New therapies have developed over the past several decades with respect to immunotherapies, particularly programmed death protein 1 inhibitors (e.g., nivolumab and pembrolizumab). Studies have shown checkpoint inhibitors to be effective in treating HL with an objective response rate of 69% for relapsed/refractory classical HL for nivolumab use. Checkpoint inhibitors will continue to help maintain the high five-year survival rate for HL and hopefully have a more favorable side effect profile in the short term, as well as later in the patient’s life. This article seeks to summarize treatment options for HL while comparing outcomes and side effect profiles with the addition of checkpoint inhibitors.

## Introduction and background

While the incidence of Hodgkin lymphoma (HL) has remained unchanged over the past 40 years, mortality rates have significantly decreased. In 1975, the incidence was 3.1 per 100,000, while the incidence in 2018 was 2.3 per 100,000. Mortality rates in 1975 were 1.3 per 100,000 which decreased to 0.3 per 100,000 in 2018 [1]. This improvement in mortality is thought to be due to advances in treatments for HL [2].

HL has a bimodal distribution with respect to age. Similar incidences in the 15-39, 65-74, and 74+ age groups (3.4, 3.6, and 3.4 per 100,000, respectively) occurred in 2017 [1]. In 2020, 89,500 new cancer cases were predicted with 9,270 cancer-related deaths in adolescents and young adults (AYAs), defined by the National Cancer Institute as patients between the ages of 15-39. Of these, 2,800 cases were predicted to be due to HL. HL incidence was found to be the highest in the 20-29 age range in one study of AYAs with an incidence of 4.1 (15-19 years and 30-39 years were 3.1 and 3.4, respectively) with a five-year survival of 95% (15-19 years and 30-39 years at 97% and 94%, respectively) using data from 2009 to 2015 [3]. HL has a five-year survival rate that has continued to increase over the last 40 years, but this success has come at a cost. Treatments increase the risk of secondary cancers for up to 40 years [4]. As HL is a disease that affects a younger population and has an excellent five-year survival rate, it is important to examine long-term treatment-related complications (including increased risk of secondary cancers, mental health disorders, and lower overall health) [5]. Of particular interest is the use of checkpoint inhibitors, such as pembrolizumab and nivolumab, in relapsed and refractory diseases. The use of checkpoint inhibitors in place of or in combination with traditional chemotherapy may help to lower the risk of developing secondary cancer due to HL treatment. This paper investigates treatment outcomes, paying particular attention to adverse effects, as well as the expanding role of immunotherapy.

## Review

Pathology and classification

HL was first described in 1832 by the pathologist Thomas Hodgkin [6]. The characteristic pathology of HL is a small number of Reed-Sternberg cells set in a background of reactive inflammation that consists of lymphocytes, eosinophils, neutrophils, histiocytes, and plasma cells. Hodgkin Reed-Sternberg (HRS) cells represent transformed B-cells in most cases. These B-cells are most likely pre-apoptotic germinal-center B-cells that have lost apoptotic mechanisms and have gained activations in multiple signaling pathways. One of these important signaling pathways is CD30 and its role in activating NFκB. It has been found that HRS cells have high expression of CD30, as do other cells in the inflammatory infiltrate of HL. It is thought that this high expression of CD30 allows for ligand-independent signaling, causing continued activation of NFκB. NFκB then translocates to the nucleus, increasing the expression of proteins involved in immune response and antiapoptotic proteins [7].

HL is subdivided into nodular lymphocyte predominant (NLPHL) and classical Hodgkin lymphoma (cHL). cHL makes up 90% of HL and behaves more aggressively than NLPHL. Further, cHL is subdivided into four subcategories: nodular sclerosis cHL, mixed cellularity cHL, lymphocyte-rich cHL, and lymphocyte-depleted cHL [2]. This review will focus on cHL.

Programmed death protein 1 in Hodgkin lymphoma

**Figure 1:**
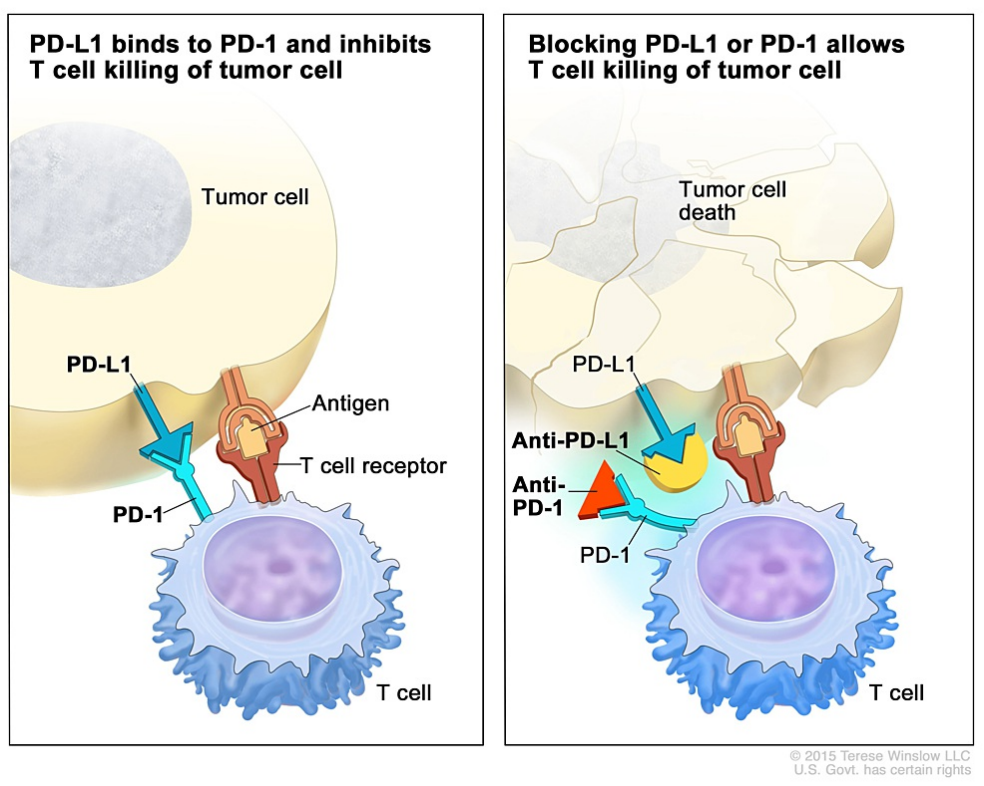
Pictorial representation of PD-L1 antibody mechanism of action. The image on the left shows PD-1 interacting with PD-L1 to cause T-cell inhibition allowing the immune system escape of the tumor cell. On the right, a PD-L1 agonist binds to PD-1 allowing for immune system destruction of the malignant T-cell. Reproduced after seeking permission from Terese Winslow LLC [14]. PD-1 = programmed death protein 1; PD-L1 = programmed death ligand 1

Programmed death protein 1 (PD-1) is an inhibitory receptor found on activated T-cells. The inhibitory role of PD-1 was shown in knockout mice that developed a lupus-like disease [8]. PD-1 interacts with PD-1 ligand (PD-L1) which can be present on tumor cells. PD-L1 can be upregulated in settings of chronic inflammation, such as chronic viral infections or cancer, as well as in the setting of chromosomal 9p24.1 amplification [9-11]. One study showed that tumor-infiltrating T lymphocytes (TILs) had higher rates of expression of PD-1 than T lymphocytes in the peripheral blood, suggesting that the tumor microenvironment plays a part in the immune response to tumors [12]. This microenvironment effect could play a major role in HL due to its highly inflammatory pathology [9]. The PD-L1 inhibitory receptor allows a pathway of escape for cancer cells from the immune system [13]. Tumor cells that express PD-L1 interact with PD-1 receptors on T-cells and inhibit activation and proliferation causing T-cells to become “functionally exhausted” [2,12]. Figure 1 helps to demonstrate this point.

Treatment

Chemotherapy

Chemotherapy has been the backbone of HL therapy since the 1960s with the introduction of the four-drug regimen of nitrogen mustard (Mustagen), vincristine sulfate (Oncovin), procarbazine, and prednisone (MOPP) [15]. In the 1970s, one cohort study compared MOPP to doxorubicin (Adriamycin), bleomycin, vinblastine, and imidazole carboxamide (ABVD) and showed that ABVD was as effective as MOPP in the treatment of HL, regardless of stage at diagnosis [16]. Studies continued to use MOPP therapy until the 1990s when it was found that ABVD was a superior regimen to MOPP or a combination of MOPP and ABVD. This combination also had lower levels of bone marrow suppression than MOPP treatment alone, as well as reduced risk of sterility [17].

Thus, ABVD was established as the standard of care for HL. This combination has been shown to be highly effective in HL, with one study reporting an 87% 12-year freedom of disease progression with ABVD use alone in limited HL [18]. However, ABVD has been associated with several undesirable effects including doxorubicin dose-dependent cardiotoxicity [19,20] as well as bleomycin lung toxicity [21].

Second-line chemotherapy regimens have been associated with adverse effects as well. One study comparing ABVD and bleomycin, etoposide, doxorubicin, cyclophosphamide, vincristine, procarbazine, and prednisone (BEACOPP) demonstrated higher adverse events in the group treated with BEACOPP. Adverse events included mucositis, severe infection, as well as grade 3 and 4 hematologic and nonhematologic adverse effects. BEACOPP may have a statistically significant higher rate of hematologic and nonhematologic adverse effects than ABVD. Hematologic adverse events were 127/156 (81%) of patients treated with BEACOPP developing grade 3 or 4 hematologic adverse effects compared to 72/166 (43%) in the ABVD-treated group (p < 0.001 between groups). A significant difference was also seen in grade 3 and 4 nonhematologic adverse effects, with the BEACOPP-treated group being 30/156 (19%) versus 12/166 (7%) in the ABVD group (p = 0.001) [22]. In the study, grade 3 and 4 hematologic and nonhematologic events were classified using the World Health Organization (WHO) Toxicity Grading Scale [23].

Radiation

Radiation therapy was first used for HL in the early 20th century with some efficacy [24]. The initial radiotherapy technique for HL was mantle field radiation [25]. Further development of techniques in radiotherapy delivery led to the development of involved-site radiation therapy (ISRT). ISRT treatment is more targeted to known sites of HL involvement and relies on chemotherapy to control potential microscopic disease that is outside of the target area [26,27]. However, radiation treatment can have side effects ranging from mild (e.g., fatigue, dry mouth, nausea, vomiting) to more severe, such as thyroid disorders, cardiovascular problems, an increased risk of chromosomal abnormalities, and secondary cancers [28-33]. Due to the increased secondary cancer risk, current radiotherapy treatments target involved lymph nodes after a short course of chemotherapy and attempts to reduce the amount of radiation exposure to uninvolved tissues [26,28,34]. However, the use of radiation may be omitted altogether. One study showed similar three-year progression-free survival (PFS) in early-stage HL (IA or IIA) patients that were positron emission tomography negative after three cycles of ABVD and then received radiation versus no further treatment (94.6% and 90.8%, respectively) [35]. The omission of radiotherapy, while still controversial, helps to prevent toxicities seen with radiotherapy and does not have a large impact on PFS in patients who are PET-negative after initial chemotherapy treatment.

Stem-Cell Transplantation

Stem-cell transplantation for the treatment of HL may be utilized for disease that has relapsed or is refractory to first-line treatment. The current standard of care for stem-cell transplantation is high-dose chemotherapy followed by autologous stem cell transplantation (autoSCT) [36]. However, there may be a role for brentuximab vedotin (BV) and PD-1 inhibitors as the first salvage treatment before autoSCT. One study examined the role of brentuximab and augmented ifosfamide, carboplatin, and etoposide (augICE). Results showed an appreciable response, with 12 of 45 patients (27%) achieving a PET-negative scan and being able to undergo autoSCT [37].

Antibody-Drug Conjugate

Antibody-drug conjugates (ADCs) have been introduced and represent a significant step forward in cancer treatment. ADC uses specific receptors to target malignant cells and deliver a cytotoxic agent while attempting to reduce systemic side effects. One ADC that is used for HL is BV. This ADC is specific for CD-30 receptors that are commonly present on Reed-Sternberg cells in cHL [38]. Scott provides an excellent review of BV in CD30-positive HL [39].

In 2011, BV was approved by the Food and Drug Administration (FDA) for the treatment of HL after the failure of autoSCT or failure of at least two prior therapies as well as systemic anaplastic large cell lymphoma after failure of at least one prior multi-agent chemotherapy regimen. BV is made up of three components, including an IgG1 antibody that is specific for the CD30 receptor, monomethyl auristatin E (MMAE) which is an antimicrotubular agent, as well as a protease-cleavable linker that combines the IgG1 and MMAE together. When IgG1 combines with the CD30 receptor on target cells, ADC internalizes into the cell. ADC internalization leads to proteolytic enzyme cleavage of the protease-cleavable linker, allowing MMAE release. The MMAE then causes tubulin disruption of the cell, leading to an arrest in the G2/M phase and apoptosis [40-42].

In a phase II study of BV for patients with relapsed or refractory HL, it was found that of the 102 patients enrolled, 94% (96 patients) showed tumor reduction. Furthermore, the primary outcome of the study was the overall objective response rate (ORR), as determined by an independent review facility using the Revised Response Criteria for Malignant Lymphoma [43]. In the study, ORR was found to be 75% (95% confidence interval (CI) = 64.9% to 82.6%) and 34% (95% CI = 25.2% to 44.4%) of patients achieved complete remission [44]. Of interest, a follow-up five-year analysis of survival from the phase II trial showed 9% (9/102) of patients achieved long-term remission in response to the sole use of BV without any other therapies [45]. Due to these promising results, a phase III trial involving 329 patients was conducted. This trial categorized the patients into two stratified groups, a group treated with BV (n = 165) and a placebo group (n = 164). An increase in PFS was seen in the group of patients receiving BV compared to the placebo group with a hazard ratio (HR) of 0.57 (95% CI = 0.40-0.81; p = 0.0013). The reported median PFS was 42.9 months in the BV group compared to 24.1 months in the placebo group. Of note though, there was no significant difference in overall survival between the two groups [45]. The most common side effects in the two trials were peripheral sensory neuropathy, neutropenia, nausea, and fatigue. The most common side effect was peripheral neuropathy, with 85% (95/112) of patients having resolution or improvement of symptoms with a median time to resolution of 23.4 weeks [44,46].

The increased efficacy that BV demonstrated in treating refractory or relapsed HL after autoSCT, while maintaining a tolerable adverse reaction profile, has made it an interesting option for further study in first or second-line treatments. Studies showed efficacy with using BV for relapsed or refractory HL before autoSCT as a single agent, combined with nivolumab (PD-1 inhibitor), or combined with a second-line chemotherapy regimen [47-49]. BV has also been studied in combination with first-line chemotherapy regimens in advanced-stage (stage III or IV) HL as well as limited-stage (stage I or II) HL [50-53]. Of note, in a phase I trial, the combination of BV and ABVD for advanced HL caused increased pulmonary toxicity over ABVD alone. Due to the increased toxicity of the combined ABVD/BV regimen, it is not advised to use BV and bleomycin in the same regimen [50]. A phase II trial examining BV + AVD for previously untreated limited-stage HL found improved efficacy, with overall survival at three years of 97%, but also a toxicity profile that was greater than would be expected with ABVD therapy [53]. Thus, BV was FDA approved in 2018 for the treatment of previously untreated advanced-stage cHL in combination with AVD but is currently not recommended in first-line treatment of limited-stage HL [54].

Immunotherapy

Immunotherapy has become a hot topic in cancer therapy in recent years, but one of its first descriptions was by Dr. William Coley in 1891. Dr. Coley injected bacterial toxins into various tumors and subsequently noted a decrease in tumor size. This decrease was presumed to be due to the immune response to the bacterial toxin that also targeted the tumor [55-57]. More recently, there have been several exciting developments in the world of cancer immunotherapy, including checkpoint inhibitors (CTLA-4 and PD-1 antibodies), chimeric antigen receptor (CAR) T-cells, lymphocyte-promoting cytokines, agonistic antibodies against co-stimulatory receptors, as well as therapeutic cancer vaccines [58,59]. Of these, checkpoint inhibitors have shown some efficacy in HL. One of the first immunotherapy FDA approvals was the CTLA-4 antibody ipilimumab in 2011 for the treatment of nonresectable metastatic melanoma [60]. Later, the PD-1 antibodies nivolumab and pembrolizumab became FDA-approved for the treatment of metastatic melanoma with progression after treatment with ipilimumab in 2014 [61,62].

In a phase I dose escalation study, the maximum dose given of nivolumab was 3 mg/kg of body weight. During the escalation from 1 mg/kg of body weight, the maximum tolerated dose was not reached [63]. This led to the usage of the 3 mg/kg dose given every two weeks in the phase II CheckMate 205 Trial. In this study, 243 patients with refractory/relapsed classic Hodgkin lymphoma (rrcHL) were assigned into three cohorts. Cohort A consisted of patients that had no prior treatment with BV (n = 63), cohort B consisted of patients who had relapsed after BV treatment and autoSCT (n = 80), and cohort C consisted of patients who had relapsed after BV treatment before or after autoSCT (n = 100). The overall ORR was 69% with a median duration of response (DOR) of 16.6 months. The most common drug-related adverse events of any grade were fatigue, diarrhea, and infusion-related reactions, with the most common grade 3 and 4 adverse events being an increase in lipase, neutropenia, and alanine transaminase increase. Also, of note, is that 44 patients in the trial proceeded to allogeneic hematopoietic cell transplant (alloSCT) after receiving nivolumab. Of these patients, 21 developed acute graft versus host disease (aGVHD). During the analysis of this small patient population, it was found that there was no significant relationship between the last nivolumab dose and grade 3 or 4 aGVHD (p = 0.97) [64]. Due to nivolumab’s efficacy and low side effect profile found in these two studies, nivolumab was FDA-approved for the treatment of rrcHL after autologous hematopoietic stem cell transplantation and post-transplantation BV in 2016 [65]. Nivolumab has also been studied in the setting of newly diagnosed advanced-stage cHL and has shown some efficacy with acceptable adverse event profiles in a small phase II trial [66].

Pembrolizumab later became FDA-approved in 2017 for the treatment of cHL that was refractory or relapsed after three or more prior therapy lines [67]. The KEYNOTE-087 trial was a single-arm phase II study of pembrolizumab in rrcHL. The study involved 210 patients split into three different cohorts, which were (1) autoSCT and then BV therapy (69 patients), (2) salvage chemotherapy plus BV (81 patients), and (3) autoSCT without BV (60 patients). These patients received 200 mg of pembrolizumab once every three weeks for a maximum of 24 months or until documented confirmed disease progression, intolerable toxicity, or investigator decision. The response was assessed every 12 weeks with a primary endpoint of ORR defined by a blinded independent central review. The ORR across all three cohorts was 69.0% (95% CI = 62.3% to 75.2%) and the complete response rate (CRR) was 22.4% (95% CI = 16.9% to 28.6%). Cohort 1 had an ORR of 73.9% (95% CI = 61.9% to 83.7%), cohort 2 64.2% (95% CI = 52.8% to 74.6%), and cohort 3 70.0% (95% CI = 56.8% to 81.2%). Each cohort had a p-value of less than 0.001 (p < 0.001). The most common treatment-related adverse events were grade 1 or 2 hypothyroidism (11.9%), pyrexia (10%), fatigue (8.6%), rash (7.6%), and headache (6.2%). The most common grade 3 events were neutropenia (2.4%), dyspnea (1%), and diarrhea (1%), with no grade 4 events being reported [68]. In a two-year follow-up to the KEYNOTE-087 study, the ORR for all cohorts was found to be 71.9% (95% CI = 65.3%-77.9%) and a CRR of 27.6%. In all responders, the median DOR was 16.5 months, with a DOR in cohort 1 of 22.1 months, cohort 2 of 11.1 months, and cohort 3 of 24.4 months. Interestingly, of patients who achieved a complete response (CR), 84.5% (n = 58) did so after greater than or equal to six months of treatment and 63.8% did so after greater than or equal to 12 months of treatment [69]. These interesting findings may suggest that patients with rrcHL may need to be treated with pembrolizumab for over a year before a CR may be seen.

As our understanding of PD-1 inhibition improves, the role checkpoint inhibitors play in the treatment of cHL may change. The KEYNOTE-204 trial compared pembrolizumab to BV in rrcHL after autoSCT or in patients ineligible for autoSCT. In total, 300 patients received treatment with either pembrolizumab 200 mg every three weeks (n = 148) or BV 1.8 mg/kg every three weeks (n = 152) for up to 35 cycles or until disease progression, unacceptable toxicity, or investigator decision. The primary endpoint for the trial was PFS, with a median PFS of 13.2 months for pembrolizumab and 8.3 in BV. The HZ for disease progression or survival was 0.65 (95% CI = 0.48-0.88; p = 0.0027). For median DOR, it was found that pembrolizumab was 20.7 months to 13.8 months with BV. Side effect profiles in the study were similar to previous trials of pembrolizumab and BV. Of note, pembrolizumab had median days on therapy of 305.0 to BV 146.5 and a median number of administrations in the pembrolizumab group of 15 to the BV group of 7. However, pembrolizumab had a longer treatment course adverse event rates were similar between the two groups, with pembrolizumab being slightly lower than BV at 74.3% of patients and 77.0% of patients experiencing adverse events, respectively [70]. This study supports the idea that pembrolizumab should be favored over BV for the treatment of cHL patients who have refractory or relapsed disease.

## Conclusions

HL is a disease that can affect a younger population as well as a disease that has had a decreasing mortality rate over the past 40 years. Stressing the importance of examining the long-term consequences of treatment. Established chemotherapy regimens, such as ABVD and BEACOPP, have been used with success in the treatment of HL, but have both short- and long-term effects. New treatments such as ADCs (brentuximab vedotin) and immunotherapies (pembrolizumab and nivolumab) have been found to be very useful in relapsed/refractory HL and have helped to revolutionize the clinical care of HL. Patients who previously would not have been able to tolerate an intense chemotherapy regime before autoSCT or who are not transplant candidates may receive these agents and the possibility of clinical improvement. Some small trials have shown potential benefits for these two treatments in previously untreated diseases, with BV+AVD now being a standard of care for newly diagnosed advanced-stage HL. The use of immunotherapies continues to grow and evolve, but more research and time are needed to determine their full utility and long-term side effects in HL.
